# Supplementary material for: Imidazolium Salt for Enhanced Interfacial Shear Strength in Polyphenylene Sulfide/Ex-PAN Carbon Fiber Composites
Source: Polymers (Basel). 2022 Sep 5;14(17):3692. doi: 10.3390/polym14173692 (PMC9459804; doi:10.3390/polym14173692)
Supplement: Supplementary file 1 [file polymers-14-03692-s001.zip › polymers-1866130-supplementary.pdf]

Supplementary Materials

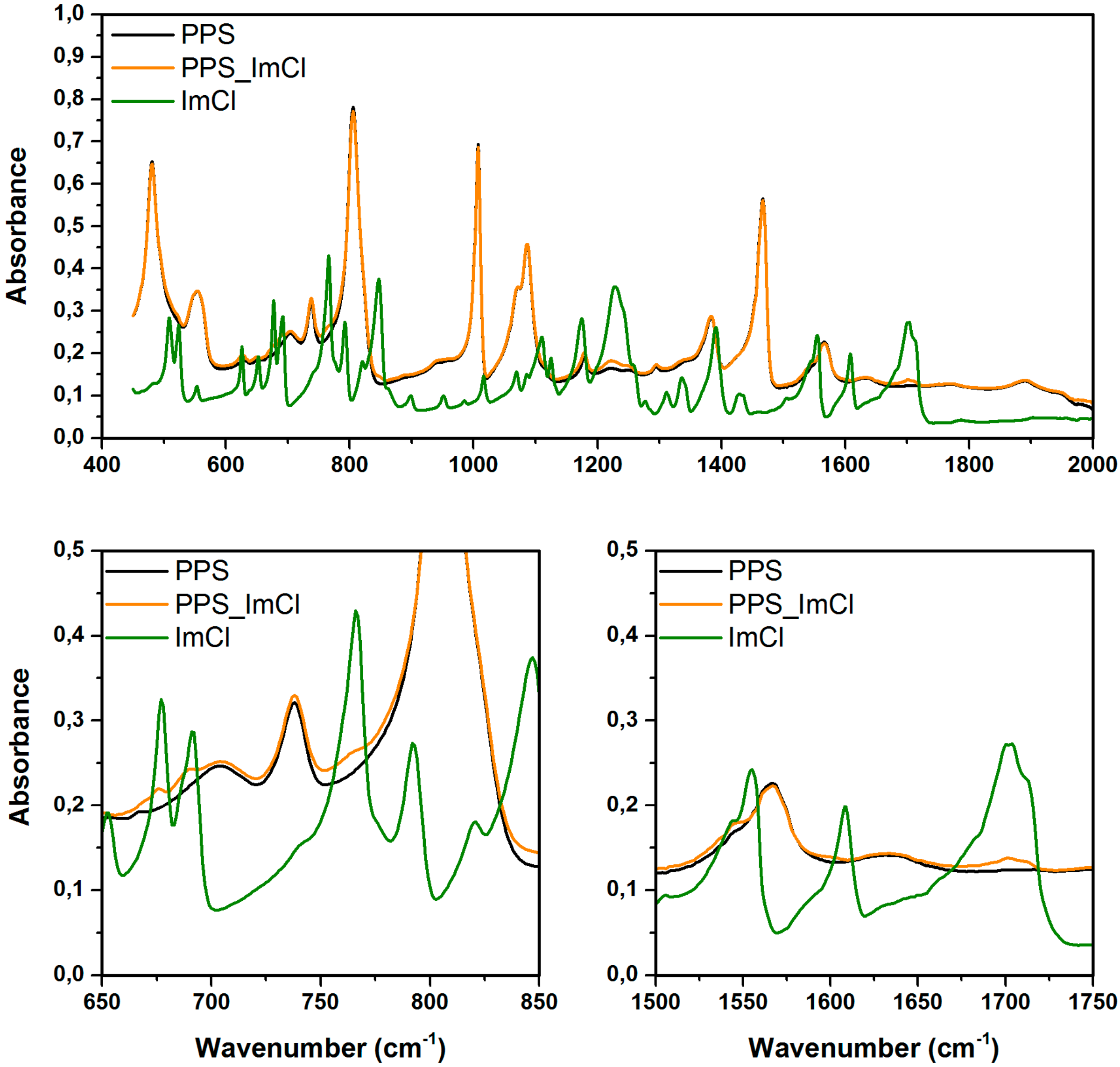

Figure S1. FTIR spectra of neat PPS ,PPS_ImCl at 300°C and ImCl at room temperature .

Table S1. Main infrared band assignments for PPS.

Peak position cm^{-1} (T_{amb}) [27]	Peak observed cm^{-1} Present work (300 °C)	Band assignment [27]
1574	1568	Sym. Ring stretching
1472	1467	Ring stretching
1389	1384	Ring stretching
1093	1086	Anti. Ring-S stretching
1074	1071	Sym. Ring-S stretching
1010	1007	C–H i.p. deformation
812	805	C–H o.p. deformation
743	738	Ring deformation
480	480	O.p. skeletal deformation

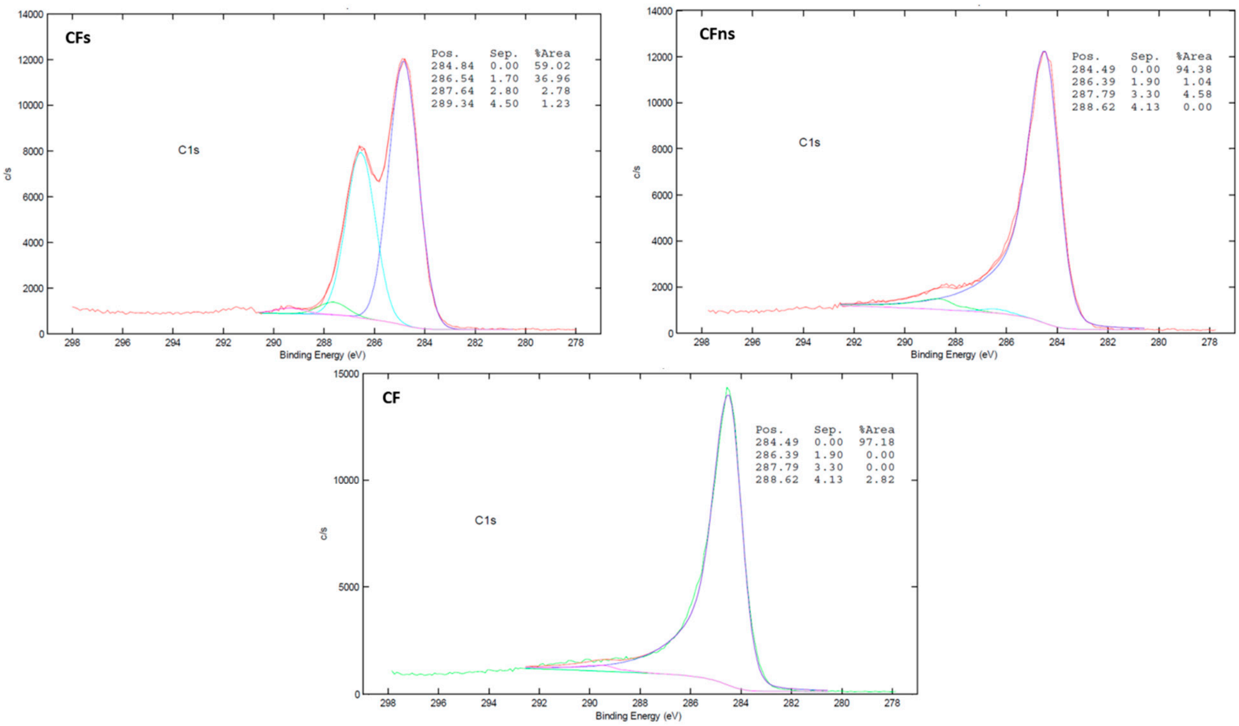

Figure S2. Decomposition of the Carbon pic.

Table S2. Chemical function obtained from the decomposition of C1s pic.

Fiber	C–C/C–H	C–O/C–N	C=O/O–C–O	O–C=O
S-CF	59	37	3	1
NS-CF	94	1	5	-
NT-CF	96	1	-	3

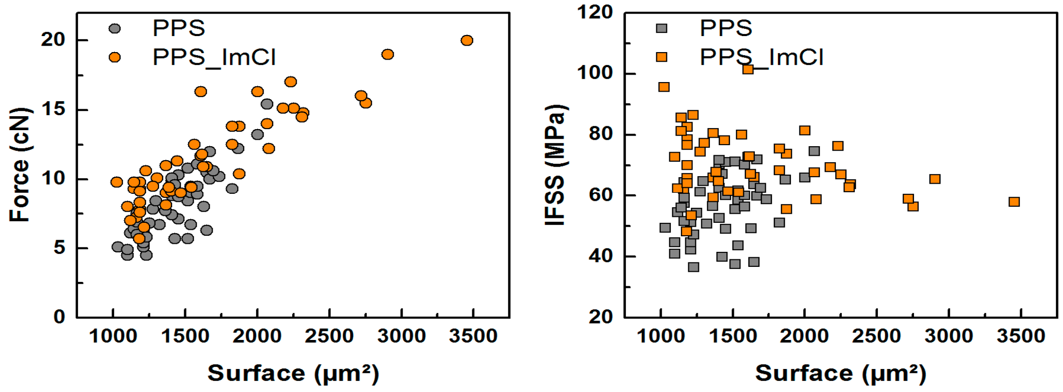

(a)

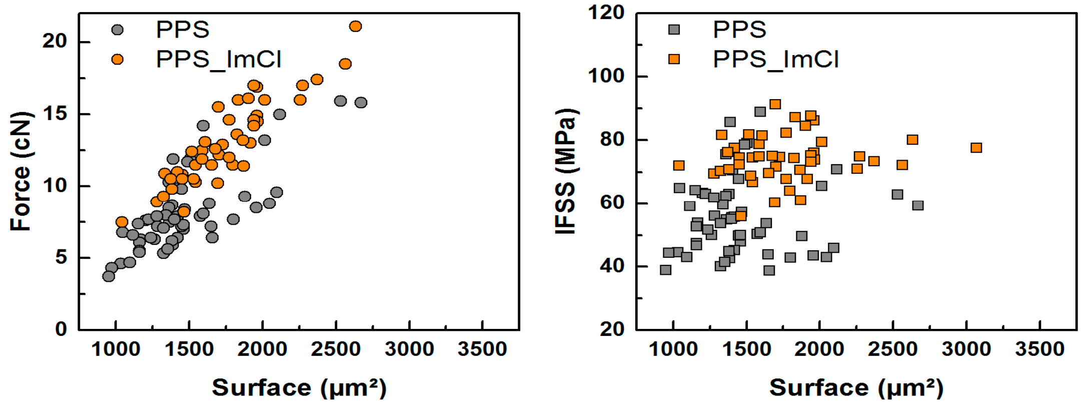

(b)

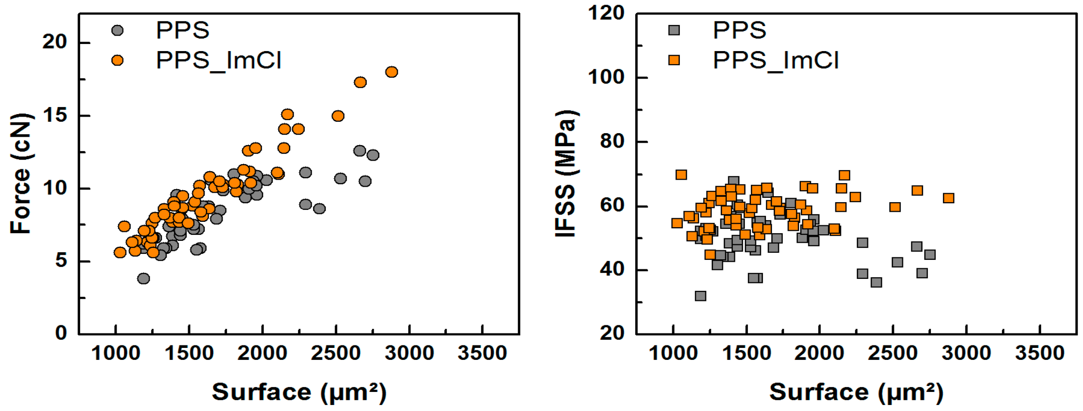

(c)

Figure S3. Evolution of interfacial Force and IFSS as a function the embedded surface for S-CF (**a**), NS-CF (**b**) and NT-CF (**c**) systems.
